# Integrating the Roles for Cytokinin and Auxin in De Novo Shoot Organogenesis: From Hormone Uptake to Signaling Outputs

**DOI:** 10.3390/ijms22168554

**Published:** 2021-08-09

**Authors:** Martin Raspor, Václav Motyka, Abdul Rasheed Kaleri, Slavica Ninković, Ljiljana Tubić, Aleksandar Cingel, Tatjana Ćosić

**Affiliations:** 1 Department of Plant Physiology, Institute for Biological Research “Siniša Stanković”–National Institute of Republic of Serbia, University of Belgrade, Bulevar Despota Stefana 142, 11060 Belgrade, Serbia; slavica@ibiss.bg.ac.rs (S.N.); tubic@ibiss.bg.ac.rs (L.T.); cingel@ibiss.bg.ac.rs (A.C.); tatjana@ibiss.bg.ac.rs (T.Ć.); 2 Laboratory of Hormonal Regulations in Plants, Institute of Experimental Botany of the Czech Academy of Sciences, Rozvojová 263, 16502 Prague 6, Czech Republic; motyka@ueb.cas.cz; 3 School of Life Science and Engineering, Southwest University of Science and Technology, Mianyang 621010, China; arasheedkaleri@yahoo.com

**Keywords:** auxin, cytokinin, de novo shoot organogenesis, DNSO, gene regulatory network, hormone uptake, shoot regeneration, sucrose, transport

## Abstract

De novo shoot organogenesis (DNSO) is a procedure commonly used for the in vitro regeneration of shoots from a variety of plant tissues. Shoot regeneration occurs on nutrient media supplemented with the plant hormones cytokinin (CK) and auxin, which play essential roles in this process, and genes involved in their signaling cascades act as master regulators of the different phases of shoot regeneration. In the last 20 years, the genetic regulation of DNSO has been characterized in detail. However, as of today, the CK and auxin signaling events associated with shoot regeneration are often interpreted as a consequence of these hormones simply being present in the regeneration media, whereas the roles for their prior uptake and transport into the cultivated plant tissues are generally overlooked. Additionally, sucrose, commonly added to the regeneration media as a carbon source, plays a signaling role and has been recently shown to interact with CK and auxin and to affect the efficiency of shoot regeneration. In this review, we provide an integrative interpretation of the roles for CK and auxin in the process of DNSO, adding emphasis on their uptake from the regeneration media and their interaction with sucrose present in the media to their complex signaling outputs that mediate shoot regeneration.

## 1. Introduction

Thanks to their totipotency, plant cells and tissues cultured in vitro are capable of regenerating into complete, fertile plants under appropriate cultivation conditions. Thus, in vitro cultures represent one of the most important tools in plant biotechnology. Plant in vitro culture techniques are rapidly evolving to optimize the efficiency of procedures in order to take full advantage of plant phenotype plasticity for agricultural, industrial or conservation purposes, as well as for applied and fundamental research. The regeneration of morphologically and physiologically true-to-type shoots from explants derived from a variety of tissues—referred to as caulogenesis or de novo shoot organogenesis (DNSO)—is being widely employed, relying on the addition of appropriate plant hormones, notably cytokinin (CK) and auxin, into the regeneration media. The amenability of plant species to shoot regeneration varies greatly, with recalcitrance to shoot regeneration in certain species presenting a major obstacle to genetic modifications for the improvement of yield, nutritional value or resistance to stress [1]. It has been reported that the targeted manipulation of CK [2] or auxin [3] signaling pathways can considerably enhance shoot regeneration, even from recalcitrant tissues.

Extensive knowledge is already available about DNSO and its hormonal regulation, but some of its aspects are constantly left out of the picture. While, for instance, the complicated signaling events related to the differentiation and spatial organization of the shoot apical meristem (SAM) have been thoroughly characterized, simple questions, such as the relationship between exogenous application of growth regulators, their uptake and effect on hormone levels in plant tissues, have not been adequately addressed. Over the last years, an array of notable review articles has offered summarization of the current knowledge on DNSO [4,5,6,7,8,9,10,11,12]. In this review, we focus on the molecular aspects of the involvement of CK and auxin in DNSO, with special emphasis on the questions on hormone uptake from the regeneration media and their crosstalk with sucrose present in the media—questions that have so far remained unanswered or poorly answered.

## 2. From the Valvekens Protocol to the Characterization of Signaling Networks

It has been known since the work of Skoog and Miller [13] that the morphogenic fate of plant tissue differentiation is affected by the ratio of CK and auxin levels in the tissue. It was however only in 1988 that the foundation for the modern two-step shoot regeneration methodology was laid, when Valvekens and co-authors published a simple and efficient shoot regeneration protocol based on the regeneration of shoots from *Arabidopsis* root explants, using a sequence of two regeneration media: a high-auxin, low-CK (2.2 μM 2,4-dichlorophenoxyacetic acid–2,4-D; 0.2 μM kinetin) callus induction medium (CIM) and a high-CK, low-auxin (25 μM *N*^6^-(Δ^2^-isopentenyl)adenine–iP; 0.9 μM indole-3-acetic acid–IAA) shoot induction medium (SIM) [14]. The protocol was superior to several other tested protocols as lower concentrations of plant growth regulators induced lower regeneration rates, whereas higher concentrations of applied 2,4-D led to the appearance of aberrant shoots and ultimately, the loss of morphogenic potential. The authors pointed out that shoot regeneration could also be obtained from root explants without pre-incubation on CIM. In such cases, however, shoot regeneration would occur only from the proximal end, not along the entire length of the root explant [14].

Ever since the publication of this work, which has, according to the Scopus database, reached more than 1000 citations to date (30 June 2021), researchers and biotechnologists around the world have used two-step regeneration protocols to obtain transgenic shoots of different plant species, adapting the protocol to the requirements of particular species. In 2002, a comprehensive transcriptomic analysis of the course of the classical two-step shoot regeneration in *Arabidopsis* was published [15,16], providing the first important insights into the molecular events that accompany the formation of calli on CIM and the start of shoot regeneration on SIM. As the foundation of our current understanding of CK signaling had been laid the previous year [17], it was possible to put the transcriptomic data into the context of both the CK signaling cascade [15] and the expression of other development-related genes [16]. Extensive molecular research over the next years revealed intricate crosstalk between the two, as well as with other signaling pathways.

## 3. The Course of DNSO: From Pluripotent Primordia to Developing Shoots

**Figure 1:**
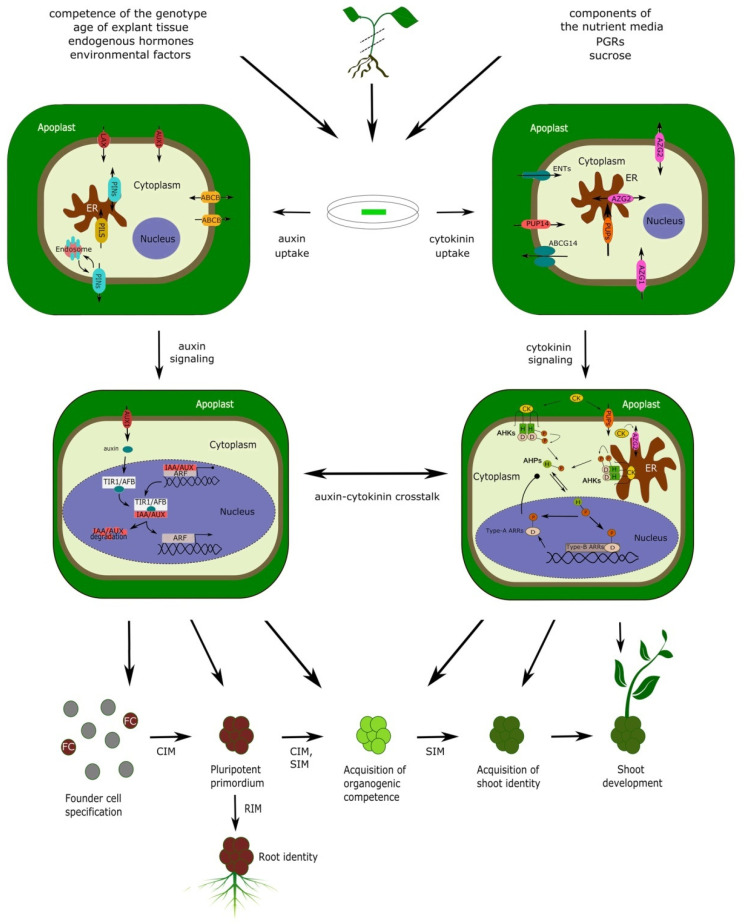
An integrative model for the roles of auxin and cytokinin (CK) in de novo shoot organogenesis (DNSO) in *Arabidopsis thaliana*. Shoot regeneration is affected by explant characteristics, such as the competence of the genotype, age of the explant tissue and its endogenous hormones, as well as by environmental conditions. Components of the regeneration media, such as exogenously added plant growth regulators (PGRs) or a carbon source (e.g., sucrose), affect regeneration efficiency. The plant hormones auxin (left side of the diagram) and CK (right side) are taken up from the regeneration medium and transported through plant tissues and into the cells. Auxin transport (upper left cell) relies, among other molecules, on auxin influx carriers AUXIN-RESISTANT1 (AUX1) and LIKE-AUX1 (LAX), efflux carriers PIN-FORMED (PIN) and transporters of the ATP-BINDING CASSETTE-B (ABCB) family. The PIN efflux carriers have a polar distribution on the plasma membrane and undergo endocytic recycling, but they are also present at the ER membrane, together with the PILS transporters. CK transport (upper right cell) relies on PURINE PERMEASEs (PUPs), EQUILIBRATIVE NUCLEOSIDE TRANSPORTERs (ENTs), ATP-BINDING CASSETTE-G (ABCG) and AZA-GUANINE RESISTANT (AZG) transporters. Once they reach their target cells, plant hormones bind to their respective receptors, triggering the corresponding signaling cascades. Auxin signalization (bottom left cell) begins when the auxin binds to the nuclear receptor TRANSPORT INHIBITOR RESPONSE1/AUXIN SIGNALING F-BOX (TIR1/AFB). The auxin-TIR1/AFB complex then releases the transcription factors of the AUXIN RESPONSE FACTOR (ARF) family from the repressive interaction with the Aux/IAA transcriptional repressors, which are then targeted for ubiquitin degradation. CK signalization (bottom right cell) starts with CK binding to the receptors of the ARABIDOPSIS HISTIDINE KINASE (AHK) family. The AHK receptors are present both on the endoplasmic reticulum (ER) and on the plasma membrane (PM). CK molecules may bind to the PM-located receptors; however, their signaling from the ER is likely more relevant. CK binding activates the receptor, causing it to dimerize and to autophosphorylate a histidine (H) residue on its protein kinase domain. A phosphorylation cascade starts, whereby the phosphate is transferred to a conserved aspartate (D) residue on the C-terminal domain of the receptor, from which it is transferred further to a H residue of ARABIDOPSIS HISTIDINE PHOSPHOTRANSFER PROTEINs (AHPs), which, upon phosphorylation, migrate into the nucleus where their activated phosphate is transferred to a D residue on the receiver domain of ARABIDOPSIS RESPONSE REGULATOR (ARR) proteins. Type-B ARRs are transcription factors that, upon phosphorylation, activate the transcription of CK-responsive genes, whereas type-A ARRs are attenuators of CK signaling that negatively regulate the upstream signaling events. Auxin and CK signaling enter into crosstalk with each other, as well as with other signaling pathways. Both signaling cascades underlie the developmental events comprising the process of DNSO, whereby the early stages of DNSO, occurring on callus induction media (CIM), are regulated by auxin, and the later ones, occurring on shoot induction media (SIM), are regulated mainly by CK.

Two-step shoot regeneration is subdivided into several stages, depending on the authors’ perspective, but is best described by a sequence of five main morphogenic processes: (1) founder cell specification (requires CIM); (2) formation of pluripotent primordia (requires CIM); (3) acquisition of organogenic competence (requires both CIM and SIM); (4) acquisition of shoot identity (requires SIM); (5) shoot outgrowth (occurs spontaneously after shoot identity is acquired) [4,9,11] (Figure 1). The formation of pluripotent primordia may or may not involve the formation of a mass of apparently disorganized plant tissue—the callus. There are various types of callus tissues in plants, but in general, calli are formed when intense cell proliferation is not followed by proper tissue patterning associated with normal morphogenic processes in plants [18,19]. Depending on the presence or absence of callus tissue, shoot organogenesis can be classified as either indirect (proceeding through the stage of callus formation) or direct (callus-independent).

Both direct and indirect shoot organogenesis proceed through a similar series of cellular and genetic events associated with the corresponding morphogenic stages, regardless of the presence or absence of visible callus tissue [20,21,22]. In either case, shoot organogenesis is dependent on the formation of primordia, containing a pre-existing population of stem cells that never cease to exist even in differentiated tissues [23]. Indeed, the long-standing assumption that DNSO requires tissue dedifferentiation in order to subsequently acquire organogenic competence has been recently disproved—DNSO has been shown to occur from xylem pole pericycle cells of *Arabidopsis* root and hypocotyl explants, involving the stage in which shoot primordia develop from structures present in calli that are similar to lateral root primordia (LRP) [24]. Calli derived from explants from above-ground organs like cotyledons and petals also regenerate shoots through primordia resembling LRP; moreover, mutants incapable of developing lateral roots are also unable to form calli on CIM, confirming that the organogenic primordia present within the calli share both structural and functional homology with LRP [25]. The same LR-like primordia are formed during direct shoot organogenesis without the proliferation of callus tissue [20]. These primordia, which, in the case of ontogenic root development grow to become normal lateral roots [26], can be induced within a certain developmental window to trans-differentiate into shoot primordia [27] (Figure 1). A population of stem cells exists within these primordia, ensuring their pluripotency and capability of developing a meristem [23] able to switch identity through a timely induction of changes in gene expression [27]. Such degree of developmental plasticity suggests that the cellular, genetic and developmental organization of LRP still shares considerable commonality with the most primitive traits of shoot development, which likely predate the modern organ identities of higher plants. Thus, although the primordia which appear in the early stages of DNSO share all the histological and genetic features of LRP and readily differentiate into root tissue, referring to them as “pluripotent primordia” or even simply “primordia” instead of “LRP” is more appropriate in the context of DNSO.

For certain plant species, either direct and/or indirect shoot organogenesis can be carried out in one step, without the need for pre-incubation on CIM. The requirement for CIM is dependent on plant species and genotype, but the same sequence of molecular events that accompany the stages of DNSO is likely to take place in competent explants regardless of whether they are physically incubated on CIM or not [22,28,29,30]. The morphogenic events that are part of DNSO occur within a unique, highly organized cellular and genetic framework, for which the presence of plant growth regulators in the regeneration media is merely a condition that enables certain developmental events to switch on. Moreover, since these morphogenic events occur within plant tissues and not inside the regeneration media, they are not directly dependent on the plant growth regulators present in the media, but on complex physiological outputs that these growth regulators exert on the incubated tissues as a whole. For instance, plant hormones need to be taken up from the media and transported through the plant tissues before they deliver a specific molecular signal that will trigger a particular morphogenic event. Additionally, the plant hormones taken up from the media will cause changes in endogenous levels of other plant hormones; it is these endogenous hormones, and not the concentration of growth regulators in the regeneration medium, that are relevant to the morphogenic events occurring in the plant tissues (Figure 1). In accordance with that, it was shown that a CK-induced elevation of local endogenous levels of auxin, rather than the application of exogenous auxin, is relevant to the efficiency of shoot regeneration [31,32].

The role of CK and auxin signalization is central to the entire process of DNSO, whereby the early stages of DNSO are dominated by auxin and the later stages by CK signaling. Taking into account the early observation by Skoog and Miller [13] that a high auxin/CK ratio stimulates the development of roots, while a high CK/auxin ratio is favorable to the development of shoot tissue, the general need for a sequence of two regeneration media can be explained by the differences in morphogenic requirements between early and late stages of DNSO. In the early stages, a high auxin/CK ratio is required not only for the development of calli but also of lateral root-like primordia within the calli; later, a high CK/auxin ratio will be required to convert the developmental fate of these primordia into taking on a shoot identity [4,9,27,28]. We show, further in the text, that indeed, the major master-regulator genes that dominate the early stages of DNSO are auxin-responsive, whereas those dominating the later stages are responsive to CK (Figure 1).

**Figure 2:**
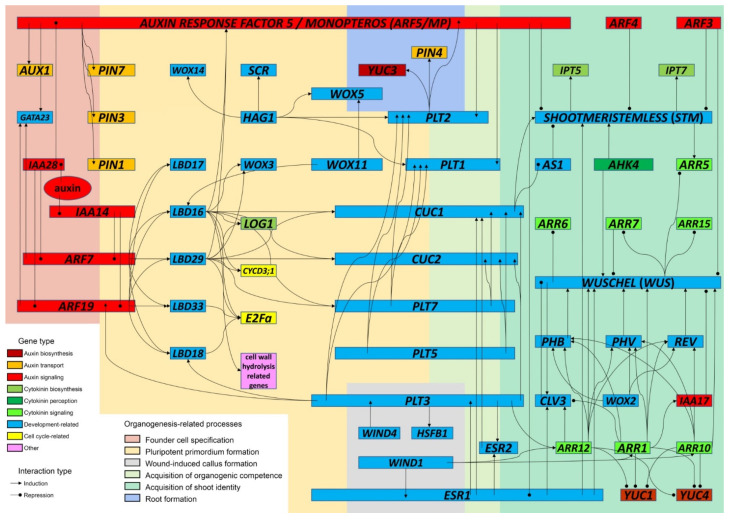
A simplified representation of the genetic regulation of the process of de novo shoot organogenesis (DNSO) in *Arabidopsis thaliana*. The process of DNSO progresses from the left side of the diagram to the right, with square boxes representing genes relevant for the control of particular stages of DNSO. Full names and brief description of the functions of the genes are provided in Table 1. The diagram does not imply temporal relationships between the expression of individual genes within a particular stage of DNSO nor that any of the genes are necessarily inactive during other stages of DNSO. Furthermore, only a selection of genes that are most relevant to DNSO and mentioned in the text are represented in the diagram. Additional interactions between genes, which are not shown in the diagram, might be in place.

The genetic basis of DNSO is extraordinarily complex and involves the participation of a tremendous number of genes, among which the most prominent include transcription factors, hormonal response regulators, genes involved in hormonal metabolism and transport, and cell-cycle-related genes (Figure 2 and Table 1).

### 3.1. DNSO Begins with the Specification of Founder Cells in the Xylem Pole of the Pericycle

The earliest morphogenic event relevant to DNSO that occurs upon the incubation of an explant on CIM, is the specification of founder cells in the xylem pole of the pericycle, which will later give rise to pluripotent primordia [4,5,9]. The equivalence with the LRP suggests that the specification of founder cells is triggered by the presence of auxin in the CIM in a similar manner as the specification of lateral root founder cells is triggered in the root basal meristem. The establishment of a dynamic gradient of auxin in the root pericycle leads to the specification of cells within local auxin maxima into founder cells [33]. The expression of auxin transporter genes—both the upregulation of auxin influx and downregulation of auxin efflux carriers—is crucial for the establishment of local auxin maxima that trigger founder cell specification. Thus, the auxin influx carrier gene *AUXIN-RESISTANT1* (*AUX1*) is expressed in the pericycle cells before the first periclinal division [34]. Conversely, auxin efflux carriers like *PIN-FORMED* (*PIN*) need to be downregulated to prevent auxin outflow during founder cell specification [35], but subsequent transient upregulation of *PIN3*, and possibly *PIN7*, is necessary for the initiation of the pluripotent primordia in the next step of organogenesis [36]. Both *PIN3* and *PIN7*, as well as *PIN1*, are inducible by the *AUXIN RESPONSE FACTOR5/MONOPTEROS* (*ARF5/MP*) [37], making it a likely candidate for the initiation of this process. This particular auxin response factor is an important regulator of multiple stages of DNSO, and it has been suggested that mutations of its C-terminal domain, releasing it from negative regulation by Aux/IAA repression, can confer improved efficiency of shoot regeneration [3].

The synthetic auxins such as 2,4-D, which is less efficiently transported out of the cells by PIN carriers, and naphthaleneacetic acid (NAA), which can circumvent the AUX1/LAX influx carriers to enter the cells, are rendered useful primarily to create robust local auxin maxima to boost the efficiency of founder cell specification in the first phase of DNSO [4].

The occurrence of local auxin maxima in the root pericycle was shown to coincide with the expression of the gene *GATA23* during lateral root formation. The transcription factor GATA23, regulated by auxin-responsive transcriptional repressor Aux/IAA28 through the inhibition of ARF5, ARF7 and ARF19, was reported to be crucial for switching on the lateral root founder cell identity [38]. Interestingly, *GATA23* was shown to be an important genetic marker for root-knot nematode-induced root galls in *Arabidopsis*, along with other auxin-responsive genes like *LATERAL ORGAN BOUNDARIES DOMAIN16* (*LBD16*), *WUSCHEL-RELATED HOMEOBOX5 (WOX5*), *SCARECROW* (*SCR*), *IAA12*, *IAA14*, *IAA28* and *ARF5/MP*, which are all known to participate in early, CIM-dependent stages of DNSO [39]. It will be interesting to see whether further transcriptomic research of the early stages of DNSO will reveal the participation of *GATA23* in the founder cell specification of pluripotent primordia on CIM.

### 3.2. Initiation of Pluripotent Primordia Relies on the Activity of Auxin-Regulated Genes LBD, WOX and PLT

To understand the molecular events associated with the formation of pluripotent primordia on CIM, more valuable cues can be drawn from analogies with lateral root development. A crucial discovery in the research on lateral root formation was the isolation of *solitary-root* (*slr*), a dominant gain-of-function mutation responsible for a peculiar phenotype in which mutant plants developed a normal primary root but were completely unable to initiate lateral roots. The mutation was localized to the gene encoding an auxin-responsive transcription inhibitor, *IAA14* [40]. Furthermore, double mutants for *ARF7* and *ARF19* displayed a similar, albeit milder phenotype, as they were able to form lateral roots but with a great delay in comparison to wild-type plants [41]. It was further shown that, in normal lateral root development, *IAA14* acts as a repressor of *ARF7* and *ARF19*, thereby inhibiting the premature formation of lateral roots during ontogenic development [42]. The auxin response factors *ARF7* and *ARF19* are activators of the initiation of the LRP, a function which they exert through the activation of transcription factors of the *LATERAL ORGAN BOUNDARIES DOMAIN/ASYMMETRIC LEAVES2-LIKE* (*LBD/ASL*) family: *LBD16*, *LBD17*, *LBD18*, *LBD29* and *LBD33* [43,44,45]. In contrast to *arf7arf19* double mutants, single *lbd16* and *lbd18* and double *lbd16lbd18* mutants were able to develop LRP but were impaired in lateral root emergence [44]. The downstream functions of the LBD transcription factors are diverse. For instance, LBD18 and LBD33 form a heterodimer which activates the expression of the cell-cycle-related gene *E2 PROMOTER BINDING FACTOR a* (*E2Fa*); the expression of *E2Fa* was shown as rate-limiting for the initiation of LRP [45].

The definite confirmation that callus formation during DNSO shares the molecular pathway with the initiation of LRP in ontogenic development came when it was shown that *LBD16*, *LBD17*, *LBD18* and *LBD29* were dramatically upregulated on CIM in *Arabidopsis* root explants, and that the ectopic expression of either of these four genes was able to induce callus formation on media without the addition of plant growth regulators [46]. The role of the ARF/LBD-activated expression of *E2Fa* was also confirmed in callus formation in *Arabidopsis* [47]. Furthermore, it was reported that the auxin-mediated induction of *LBD29* in *Arabidopsis* activates an array of various genes leading to callus formation, most importantly those related to ROS metabolism, lipid metabolism, cell wall hydrolysis and methylation [48]. A basic region/leucine zipper motif transcription factor, bZIP59, was shown to be important for LBD16-induced callus formation in *Arabidopsis*. CIM incubation enhanced the formation of the complex bZIP59-LBD16, which acted as a transcriptional activator of *FAD-Binding Berberine* (*FAD-BD*), a gene encoding an enzyme of cell wall metabolism, which is important for callus formation [49]. Besides being activated by the *ARF7/ARF19* system, *LBD16* can be activated by *WUSCHEL-RELATED HOMEOBOX11* (*WOX11*), which is, in its turn, also positively regulated by auxin [50]. Similarly to the *ARF7/ARF19*-mediated activation of *LBD16*, the pathway involving *WOX11* also results in the formation of calli, whereby *LBD16* is responsible for the primordium cell identity and the promotion of cell divisions during callus initiation [51]. It was shown that, during ontogenic development, with the differentiation of the primordium into an emerging root, the *WOX11*-activated expression of *LBD16* ceases and is replaced by the expression of *WOX5* and *WOX7*, which are markers of the root apical meristem [52].

It has been proposed that the continuous formation of calli on CIM is enabled thanks to the large quantities of exogenous auxin in the media, which continuously stimulates the formation of new primordia and prevents their differentiation into roots. In contrast, homeostatic mechanisms operating in plants during normal root development are able to direct the local distribution of endogenous plant hormones (mainly auxin) in order to allow for the morphogenic switch from *LBD16*-dominated to *WOX5/WOX7*-dominated, triggering the differentiation of root tissue [51].

The role of *WOX5* in ontogenic development consists in maintaining the stem cell pluripotency and proliferation in the root stem cell niche [53]. WOX5 inhibits the expression of *CDF4*, coding for a differentiation factor, in the root columella cells, which keeps them undifferentiated and maintains the population of stem cells in the root meristem [54]. The expression of *WOX5* in the root apical meristem is regulated by auxin, although differentially depending on cell type [55]. In the context of DNSO, *WOX5* was shown, together with several other genes coding for transcription factors (*WOX14*, *SCR*, *PLETHORA1*, *PLETHORA2*), to undergo epigenetic activation through histone acetylation by the histone acetyltransferase HAG1, which facilitates their transcriptional activation for the formation of the pluripotent primordia. The *wox5* and *wox14* single-gene mutants showed defects in root growth and shoot regeneration efficiency, while the triple *wox5wox7wox14* mutants displayed a more severe phenotype. Similarly, double *wox5scr* mutants displayed lower shoot regeneration efficiency and a notable downregulation of *PLETHORA1* and *PLETHORA2*. Moreover, while the expression of *WOX5* and *SCR* during the incubation of *Arabidopsis* explants on CIM enhanced the subsequent shoot regeneration efficiency, their prolonged expression during SIM incubation had an inhibitory effect, suggesting that the expression of these genes plays a role in the early phases of organogenesis, most likely in the maintenance of pluripotency. It has been suggested that, during shoot regeneration, the role of *WOX5* in the acquisition of pluripotency might be to maintain the population of undifferentiated, pluripotent stem cells for the subsequent development of primordia, similarly to its role in ontogenic root development [56]. In analogy to that, *SCR* was shown to negatively regulate ARABIDOPSIS HISTIDINE KINASE3 (AHK3)-ARABIDOPSIS RESPONSE REGULATOR1 (ARR1)-dependent CK signalization and subsequent downstream auxin biosynthesis, as part of its function in maintaining the root stem cell niche [57], which makes its potential role in the early phases of shoot regeneration even more intriguing.

*PLETHORA* (*PLT*), a family of transcription factors belonging to the *APETALA2/ETHYLENE RESPONSE FACTOR* (*AP2/ERF*) class, are extensively involved in crosstalk with auxin metabolism and signaling [58]. For instance, *PLT1* and *PLT2* are regulated by the auxin response factor ARF5/MP [58], whereas *PLT2* induces the auxin biosynthesis gene *YUCCA3* (*YUC3*), the auxin transporter *PIN4* and the same auxin response factor *ARF5/MP* in the root apical meristem [59]. Key roles for *PLT3*, *PLT5* and *PLT7* have been shown in multiple stages of DNSO, from primordium formation to the acquisition of shoot identity [60]. The expression of these three genes is not necessary for callus formation; however, the correct development of the LRP, as well as pluripotent primordia within the callus, depends on their expression. During DNSO in wild-type *Arabidopsis* explants, *PLT3*, *PLT5* and *PLT7* were upregulated by auxin in the callus tissue on CIM, as well as around shoot progenitors during the incubation on SIM until shoots were formed [60].

#### Wound-Induced Callus Formation Employs Signaling Pathways Distinct from Those Induced on CIM

In most cases, indirect shoot regeneration is carried out from explants, which represent cut segments of various plant parts, such as roots, hypocotyl or cotyledons. After several days of growth on CIM, calli are visible along the cut edge of the explant, corresponding to the surface of the wounded tissue. Expression analysis revealed the strong induction of four genes of the *AP2/ERF* class upon wounding; they were named *WOUND-INDUCED DEDIFFERENTIATION1* (*WIND1*), *WIND2*, *WIND3* and *WIND4* [61]. Ectopic expression of each of these genes was able to induce intense cell proliferation and callus formation without the need for exogenous growth regulators. However, quadruple *wind1wind2wind3wind4* mutants could still form calli upon wounding, suggesting that the expression of *WIND* genes is not the sole mechanism of wound-induced callus formation. The capability of ectopic expression of *WIND1* to induce calli in the *solitary-root* mutants incapable of developing LRP further suggested that *WIND1*-induced callus tissue is qualitatively different from calli induced on CIM. A surprising finding was that *WIND1* induces callus formation through the activation of CK and not auxin signaling [61]. Mutants defective in CK synthesis displayed reduced efficiency of wound-induced callus formation, suggesting that the de novo synthesis of CK might be important for this type of response [62]. In response to wounding, *WIND1* acts as a transcriptional activator of *DORNRÖSCHEN/ENHANCER OF SHOOT REGENERATION1* (*DRN/ESR1*), which activates both wound-induced callus formation and shoot regeneration. Although not completely abolished, shoot regeneration was considerably impaired in *esr1* mutants [63].

A comprehensive analysis of gene regulatory networks confirmed the existence of two separate but somewhat overlapping pathways of callus formation in *Arabidopsis*, one of which is induced by incubation on CIM and the other by wounding [21]. The analysis revealed that the key regulatory nodes of the gene regulatory network involved in the cellular reprogramming associated with the acquisition of pluripotency were *PLT3*, *ESR1* and *HEAT SHOCK FACTOR B1* (*HSFB1*). Among those, *PLT3* and *ESR1* could be induced by either wounding or incubation on CIM; *HSFB1* was wound-induced but not CIM-induced, while other important regulatory nodes included *CUP-SHAPED COTYLEDON2* (*CUC2*) and *WUSCHEL* (*WUS*), which were CIM-induced but not wound-induced. Interestingly, *PLT3* was regulated by *ESR1*, *ASYMMETRIC LEAVES2* (*AS2*) and *WIND4* but in its turn regulated a diverse array of downstream targets, including *LBD18*, *HSFB1*, *ESR2*, *CUC1*, *WIND2* and *WIND3*, an auxin response factor (*ARF19*), a CK response factor (*ARR12*), two inhibitors of cyclin-dependent kinases (*KRP2* and *KRP3*) and several other genes encoding transcription factors. At the same time, an array of *LBD* transcription factors (most importantly, *LBD16* and *LBD29*) regulated a set of target promoters mostly distinct from the targets of *PLT3*, including: the transcription factors *PLT7*, *CUC1*, *CUC2* and *WOX3*; the cell-cycle-related genes *CYCLIN D3;1* (*CYCD3;1*) and *E2Fa*; the auxin response factor *ARF5/MP*; the CK biosynthetic gene *LONELY GUY1* (*LOG1*) and several other genes [21]. The complexity and intricacy of the described gene regulatory network is such that it is impossible to single out particular roles for CK, auxin or wound-induced signaling in the process of cellular reprogramming and the acquisition of pluripotency.

### 3.3. Both Auxin and CK Are Needed to Induce the Acquisition of Competence for Shoot Regeneration

Once the pluripotent primordia are formed, their further path to shoot regeneration depends on their competence for organogenesis. In the two-step shoot regeneration protocol, the acquisition of organogenic competence starts on CIM and continues after the explants are transferred to SIM. The reliance of organogenic competence on both auxin and CK adds complexity to the interpretation of the intricate regulatory interactions that underlie DNSO, blurring the borders between the stages of the process. Furthermore, the effects of individual groups of genes cannot always be clearly confined to a particular stage of the process; hence, in the representation of the course of DNSO, various authors ascribe the contribution of particular groups of master regulator genes to different stages of DNSO, with noteworthy differences in the interpretation of the acquisition of competence for shoot regeneration [4,7,9,11].

On the genetic level, the acquisition of organogenic competence consists of switching on a series of regulatory circuits resulting in the preparation of pluripotent primordia for morphing into a shoot promeristem [4,7,9]. The ability of calli to turn green has been traditionally interpreted as an indicator of their organogenic competence; however, a recent screening of *Arabidopsis* accessions showed insufficient correlation between the ability of calli to produce chlorophyll and their competence to regenerate shoots [64].

A pioneering study on the acquisition of organogenic competence was carried out in a direct regeneration system with *Arabidopsis* LRP as starting material. Using adequate phytohormone treatment, it was possible to induce the transdifferentiation of the organ identity of the developing primordia, and it was even possible to reverse it back and forth but only within a narrow developmental window [27]. For instance, in stage VI or VII Col-0 *Arabidopsis* root explants, it was possible to efficiently induce transdifferentiation into fully functional shoot primordia through the application of *N*^6^-(Δ^2^-isopentenyl)adenine (iP). However, younger or older root explants were not competent for transdifferentiation; competence for transdifferentiation was found to coincide with the existence and size of a *WOX5*-expressing domain, which corresponds to the root stem cell niche. Cell divisions arrested for about 30 h upon transfer to CK-containing media, after which they resumed, giving rise to a primordium with altered identity. The activity of auxin and CK signaling reporters confirmed the transition of signaling profiles from root-primordium-like to shoot-primordium-like identity. Gene expression analysis also revealed the transition of root primordium expression profiles to those typical of the SAM, with the rapid downregulation of *PLT1* and *SHORTROOT* and the establishment of *SHOOTMERISTEMLESS* (*STM*), *WUSCHEL* (*WUS*) and *CLAVATA3* (*CLV3*) expression patterns typical of the developing SAM. About 3000 genes in total, among which transcription factors were most numerous, were differentially expressed during the identity switch. The shoot primordia could also, within a short developmental window, be reverted to the root primordium identity through treatment with NAA; these changes were accompanied by the restoration of a phytohormonal and expression profile typical of the root primordium. It has been demonstrated that the rapid identity switch is carried out through simple switches between equivalent elements of a developmental program: for instance, the same stem cell niche switches identity between root stem cell niche and SAM; or, *WOX5* expression in the root stem cell niche is quickly replaced with the equivalent expression pattern of *WUS* to assign a shoot identity to its expression domain [27].

Accumulated evidence suggests that the key master regulator genes of the acquisition of organogenic competence belong to the families *PLT*, *ENHANCER OF SHOOT REGENERATION* (*ESR*) and *CUP-SHAPED COTYLEDON* (*CUC*), all of which depend on both auxin and CK to exert their functions in DNSO and act upstream of the key master regulator genes of the SAM.

As stated earlier, versatile roles have been reported for *PLT3*, *PLT5* and *PLT7* in multiple stages of DNSO, depending on activation by both CIM and SIM [60]. These three genes activate *PLT1* and *PLT2*, which are responsible for the establishment of shoot progenitor cell lines, a prerequisite for organogenic competence. In the *plt3plt5plt7* triple mutants, the ability to form calli was unaffected, but the plants produced aberrant LRP, suggesting that shoot regeneration from primordia in the callus might also be compromised; indeed, callus greening and the formation of shoot promeristem were abolished, although they could be restored through the activation of *PLT1* and *PLT2* in the *plt3plt5plt7* mutant background. However, the expression of *PLT1* and *PLT2* could not rescue the shoot regeneration in the *plt3plt5plt7* mutants, suggesting that *PLT3*, *PLT5* and *PLT7* are also responsible for later stages of shoot regeneration. Indeed, it was shown that these genes are necessary for the (probably direct) activation of *CUC1* and *CUC2*, as well as for the correct expression patterning of the WUS-CLV3 circuit in the SAM. Shoot regeneration could not be rescued by the expression of *WUS* or *ESR2* in the triple mutant background. Moreover, in the calli of *plt3plt5plt7* triple mutants, both PIN1-mediated auxin redistribution and auxin signaling were remarkably compromised, suggesting that the molecular processes induced by PLT might be heavily relying on downstream auxin signaling. However, the reconstitution of PIN1 alone was not sufficient to abolish the failure to regenerate shoots. Additionally, in wild type *Arabidopsis* plants, *PLT3*, *PLT5* and *PLT7* were upregulated around the primordia during direct shoot organogenesis (without the callus phase), as well as on SIM lacking auxin, suggesting that (1) *PLT3*, *PLT5* and *PLT7* can be also induced by CKs in the SIM; and that (2) their activity is required for shoot formation regardless of the composition of the regeneration media [60].

*ESR1* is another *AP2/ERF*-class gene, first identified because its overexpression was able to confer CK-autonomous shoot regeneration but also a multiple-fold enhancement of shoot regeneration efficiency in the presence of CK [65]. *Arabidopsis* plants carrying *DORNRÖSCHEN* (*drn*), an insertional mutation in *ESR1*, were characterized by the premature arrest of SAM activity due to the deregulation of the key master regulators of SAM formation: *STM*, *WUS* and *CLV3* [66]. A recent report revealed that the activity of *ESR1* is regulated by auxin in the SAM, as an auxin response gene, *ARF5/MP*, acts as its transcriptional repressor [67]. A comprehensive gene regulatory network study revealed *ESR1* as one of the most important regulatory nodes in the process of DNSO, acting upstream of *PLT3* and sharing many of its regulatory targets, whereas *PLT3* appears to act upstream of *DORNRÖSCHEN-LIKE/ENHANCER OF SHOOT REGENERATION2* (*DRNL/ESR2*), a paralog of *DRN/ESR1* [21]. *PLT3* is the “missing link” in the indirect regulation of *DRNL/ESR2* by *DRN/ESR1,* which was observed earlier [68], but *DRNL/ESR2* is probably also directly regulated by *DRN/ESR1* [21]. The expression of *DRN/ESR1* is already present in the pluripotent primordia, and it appears to be a marker of their competence to transdifferentiate into shoot primordia. *DRNL/ESR2* is, however, expressed only in a later phase, during the development of the shoot promeristem [68]. Both *DRN/ESR1* and *DRNL/ESR2* are positive regulators of *CUC1* [69,70] and of the G1-S cell cycle transition in the SAM [71]. They are both key regulators of SAM initiation, as *esr1esr2* double mutants displayed drastically reduced shoot regeneration efficiency [68].

*CUC1*, *CUC2* and *CUC3* are partially redundant genes that code for NAC-domain transcription factors involved in the formation of the boundary region between cotyledons [72,73]. Single mutations in *CUC* genes do not affect the plant phenotype; however, homozygous double *cuc1cuc2* and triple *cuc1cuc2cuc3* mutants develop a single, fused, cup-shaped cotyledon, fail to develop the SAM and are difficult to regenerate in vitro [72,74,75]. Although they were identified at first for their role in defining the organ boundary domain, *CUC* genes appear to be regulatory genes of broader importance for shoot development, and they function as upstream regulators of the SAM-related morphogenic gene *STM* [74,76,77]. Analysis of gene expression during shoot regeneration from *Arabidopsis* root explants has shown that *CUC* genes are upregulated much before shoot commitment, while root explants were still on CIM, prior to the upregulation of the main shoot morphogenic genes such as *WUS*, *STM* and *CLV1* [16,78]. *CUC1* positively regulates SAM formation not only through *STM* but also through an *STM*-independent pathway that is negatively regulated by the transcription factor *ASYMMETRIC LEAVES1* (*AS1*), as *35S::CUC1* plants do develop adventitious SAMs even in a double mutant *as1/stm* background [77]. *CUC1* is considered a genetic marker of the SAM progenitor cells during SAM formation, whereas it is replaced by *STM* expression concomitantly with the differentiation of the SAM progenitor cells into SAM cells [79]. Similarly, *CUC2* is expressed in broad areas of callus tissue during the incubation on CIM, whereas upon transfer to CK-containing SIM, its expression is confined to domains coinciding with PIN1-dependent local auxin maxima, giving space to the establishment of *WUS* expression domains dominated by CK signals. This leads to the partitioning of cell identity between progenitor and non-progenitor cells and finally, to the establishment of foci of shoot regeneration—the greening groups of cells within the callus from which individual shoots will be regenerated [78]. A study involving calli of different *Arabidopsis* genotypes (wild type, *cuc1* and *cuc2* single and double mutants, and *CUC1* and *CUC2* single and double overexpressors) showed that the regeneration capacity of a given genotype was independent of the hormone formula of SIM. Thus, *CUC1* and *CUC2* regulate organogenesis downstream of CK stimulation; however, in all genotypes, previous incubation on CIM was necessary to allow organogenesis [74].

### 3.4. Formation of the Shoot Apical Meristem Is the Key Event in the Acquisition of Shoot Identity

A primordium that has become competent for shoot organogenesis can acquire morphological and functional features of the shoot promeristem and subsequently, the meristem [4,5,78]. Although differences exist between the formation of the SAM in ontogenic shoot development and DNSO, these processes are largely similar on the genetic level [4,16,80].

The role of the two homeodomain transcription factors *STM* and *WUS* is central to the process of SAM formation. *STM* and *WUS* act independently from each other; however, their joint action is required for SAM formation [4]. The activity of both *STM* and *WUS*, as well as their regulation, are strongly interconnected with CK signaling. Both *STM* and *WUS* are upregulated by CK, consistent with the remarkable increase of their transcript levels following the transfer of calli from CIM to SIM [16]. Accordingly, the transcription of *STM* is enhanced in CK-overproducing *Arabidopsis* plants [81]. The activity of *STM* is dependent on the CK receptor *ARABIDOPSIS HISTIDINE KINASE4* (*AHK4*) [82], which is, in turn, induced by auxin pretreatment during the acquisition of organogenic competence on CIM [83]. However, auxin itself suppresses the expression of *STM* through negative regulation by auxin response factors ARF3/ETTIN, ARF4 and ARF5/MP, enabling the differentiation of cells necessary for the formation of lateral organs in the peripheral zone of the SAM [84]. *WUS* is also upregulated by the CK present in the SIM, requiring the previous induction of the CK receptor *AHK4* by auxin pretreatment during the acquisition of organogenic competence [83].

*STM* is a transcription factor of the *KNOTTED1-LIKE HOMEOBOX* family, which promotes cell division and suppresses cell differentiation in the SAM. It is believed that *STM* plays a key role in switching on shoot commitment, as shoot regeneration becomes SIM-independent after the expression of *STM* has been initiated [85]. The expression of *STM* is ubiquitous in the SAM during meristem formation, except for the emerging primordia, where it is downregulated [4]. In embryogenic SAM formation, the strong expression of *STM* is necessary for the establishment of the boundary domain—the ring of cells that separates the slow-growing stem cells in the center of the SAM from the fast-growing peripheral zone [86]. While a local auxin minimum, reliant on PIN-mediated auxin efflux from the boundary domain, is usually a prerequisite for the establishment of the boundary and the consequent expression of *STM* [86], it was recently shown that the expression of *STM* in the boundary domain can also be induced by mechanical signals in an auxin-independent manner [87]. Further studies confirmed that the expression of *CUC3*, but not *CUC1*, can be strongly induced in the boundary domain by the ablation of the central part of the SAM, opening the possibility that the regulation of *STM* expression by mechanical cues is mediated through *CUC3* [88]. *STM* also affects CK homeostasis through the induction of CK biosynthesis genes *ISOPENTENYL TRANSFERASE5* (*IPT5*) and *IPT7*, while at the same time upregulating the CK response regulator gene *ARR5*, which is a negative regulator of CK signal transduction [82,89].

*WUS* is a member of the *WUSCHEL-RELATED HOMEOBOX (WOX)* family, which also includes 14 other genes in *Arabidopsis* (*WOX1*-*WOX14*) that are involved in various developmental processes in plants [90,91]. They represent an old phylogenetic group of regulatory genes that diverged before the diversification of vascular plants, with highly conserved regulatory elements suggesting that their regulation by auxin and CK is ancient [92]. *WUS* suppresses cell differentiation in the SAM independently from *STM* [93,94]. The *WUS*-mediated repression of the differentiation of stem cells in the SAM relies on the histone acetylation of the loci containing auxin-responsive genes, thereby silencing auxin signaling [95]. Furthermore, *WUS* enhances tissue sensitivity to CK through the suppression of the negative regulators of CK signaling [96]. *WUS* also activates the transcription of *CLV3*, encoding a peptide ligand that, upon binding to the CLV1/CLV2 receptor kinase complex, acts as a transcriptional repressor for *WUS* outside the organizing center (OC) of the stem cell niche, thereby restricting the action of *WUS* to the OC and enabling cell differentiation outside of it [97,98,99,100]. Both mathematical modeling and experimental evidence have shown that the role of the *WUS/CLV* interplay in pattern formation in the central zone of the SAM is dependent on CK perception through AHK4 and downstream signaling [101]. The role of iP-type CKs is particularly important in AHK4-dependent *WUS* activation, as the biosynthesis of these CKs is proportionately more upregulated compared to other CK types upon *WUS* induction by SIM [28].

In April 2017, a report was published revealing that four type-B CK response regulators (ARR1, ARR2, ARR10 and ARR12) act as positive regulators of *WUS* during SAM formation in DNSO [102]. It was shown that incubation on SIM induces the removal of a repressive histone methylation from the *WUS* locus, after which the four CK response regulators activate the HD-ZIP III transcription factors PHABULOSA (PHB), PHAVOLUTA (PHV) and REVOLUTA (REV), which initiate the transcription of *WUS* [102]. Two months later, four other reports appeared almost simultaneously, providing similar results [103,104,105,106]. ARR10-binding sites were found in the promoter sequence of *WUS* and its transcriptional activation by ARR10, leading to enhanced shoot regeneration efficiency, was confirmed [103]. These findings were in accordance with previous reports that transgenic expression of *ARR10* can enhance shoot regeneration efficiency in *Arabidopsis* [2]. Furthermore, a positive regulatory role was confirmed for *ARR12* in inducing the SAM formation through the regulation of *WUS*; *arr12* mutants were strongly impaired in shoot regeneration, CK responsiveness and *WUS* expression, whereas *ARR12* overexpressing lines showed enhanced shoot regeneration [104]. Similarly, the activation of *WUS* by ARR1, ARR2, ARR10, ARR11 and ARR12 was shown in the development of axillary meristems [105]. Besides being transcriptional activators of *WUS*, three type-B ARRs (ARR1, ARR10 and ARR12) were shown to act as positive regulators of WUS during SAM formation also through the transcriptional repression of the auxin biosynthetic genes *YUC1* and *YUC4* [106]. The spatio-temporal expression pattern of *ARR1*, *ARR10* and *ARR12*, recorded in reporter lines, confirmed the induction of these genes starting from day 2 on SIM, with induction areas gradually shrinking to encompass only the central areas of the meristems within the developing primordia by day 12. Additionally, *YUC1* and *YUC4* reporter lines imaging showed that the expression of these genes was inhibited in the areas corresponding to OCs where the expression of *ARR1*, *ARR10* and *ARR12* was high. It was concluded that the repression of auxin biosynthesis inside the OC is required to maintain high expression levels of *WUS*, as it was previously shown [107] that some of the auxin-responsive genes, such as *ARF3/ETTIN*, negatively regulate *WUS*. The authors noted an interesting difference between their results concerning DNSO and the previously published reports on the molecular events during the embryogenic development of the SAM: although CK signaling acts upstream of *WUS* during DNSO, that is not the case in embryogenic development, wherein the CK signaling cascade appears to switch on much later than *WUS* expression [106]. An important circumstance that differentiates DNSO from the embryogenic development of the SAM is the presence of exogenous growth regulators—notably CK—in the regeneration media. It has been suggested before that both the phytohormone content of the explants and the expression of CK-responsive genes can be triggered by the CKs present in the regeneration media [28,29,108]. Because CK is already present in the regeneration media and being taken up by the explants, it can trigger the expression of *WUS* more efficiently compared to the factors that act upstream of *WUS* during embryogenic development—hence, during DNSO *WUS* is being induced by CK rather than the other way around.

Although the regulation of *WUS* by B-type ARRs appeared redundant and relatively simple at first, recent research revealed that their roles are much more heterogenous, even antagonistic [109]. In fact, as many conclusions of the initial research were drawn from *arr1arr10arr12* triple mutants, similar roles in the regulation of *WUS* were deduced for all the three genes. However, more detailed analysis of the individual roles of these genes revealed that ARR12 is the central positive regulator of *WUS*-mediated shoot regeneration, whereas ARR1 acts as its competitive inhibitor and attenuates the effects of this positive regulation. Specifically, ARR1 was shown to compete with ARR12 for binding to the promoter regions upstream of *WUS* and *CLV3*. The competitive binding of ARR1 to the *CLV3* promoter represses its ARR12-mediated transcription, while its binding to the *WUS* promoter enhances the transcription of *WUS* in non-target cells, thereby delaying the formation of the intercellular transcript gradient. The transcriptional activation of *IAA17*, an auxin-response gene that acts as a transcriptional inhibitor of *WUS*, represents another inhibitory effect of ARR1 [109].

Besides being regulated by B-type ARRs, *WUS* also increases the sensitivity of the meristem tissue to CK through the transcriptional repression of A-type ARRs, known as attenuators of CK signaling. Thus, *WUS* negatively regulates *ARR5* (which is, on the contrary, upregulated by *STM*), along with *ARR6*, *ARR7* and *ARR15*. Furthermore, *ARR7* is a negative regulator of *WUS* [96]. Accordingly, both *ARR7*- and *ARR15*-overexpressing *Arabidopsis* plants exhibited suppressed shoot regeneration, while loss-of-function mutations in these genes conferred enhanced callus formation and shoot regeneration [110]. A recently published, comprehensive gene regulatory network analysis revealed the convergence of B-type ARRs towards regulating both *WUS* and the A-type ARRs, underlining the importance of CK signaling in the regulation of shoot development [111].

During embryogenic SAM formation in *Arabidopsis*, *WOX2*, rather than *WUS*, is responsible for initiating the establishment of stem cell progenitor lines in the stem cell niche, as well as the subsequent maintenance of their pluripotency through fine-tuning the balance between auxin and CK signaling in the SAM. The promotion of CK activity in the SAM at least partially relies on the WOX2-mediated upregulation of the HD-ZIP III transcription factors PHB, PHV and REV, while local auxin concentration is kept at low levels by means of enhanced basipetal transport through the upregulation of *PIN1* [112]. *WOX2* plays also other roles in the regulation of shoot development, such as lateral organ formation and separation [113]. Similarly to its role in embryogenic development, *WOX2* seems to play an important role in establishing the SAM during DNSO, as either *WOX2/WOX8* or *WOX2/WOX9* co-expression could result in successful shoot organogenesis from transgenic tobacco leaves on media containing auxin, without the need for exogenous CK [114]. The *WOX*-mediated promotion of CK signalization occurs on multiple levels, as the *WOX2/PHB/PHV/REV* network likely involves the upregulation of *IPT1* during stem cell initiation in *Arabidopsis* [112]. Furthermore, overexpression of *STENOFOLIA*, an *AtWOX1* ortholog from *Medicago truncatula* in the energy crop switchgrass (*Panicum virgatum*), contributed to elevated CK levels through the repression of *CYTOKININ OXIDASE/DEHYDROGENASE* (*CKX*) genes [115].

The acquisition of shoot identity and the formation of the SAM, regulated by both auxin- and CK-dependent genes, are predominantly viewed as the fourth and last step in the morphogenic pathway of DNSO. Even though a fifth phase (i.e., shoot outgrowth) is commonly described as part of DNSO (Figure 1), it is actually identical to the process of shoot growth that occurs during normal plant ontogenic development and proceeds spontaneously in cultured plants even when they are transferred to growth-regulator-free media [4].

## 4. Sucrose Interferes with Auxin and Cytokinin Signaling in the Regulation of Shoot Organogenesis

During their in vitro development, plant shoots express a low capability for photosynthesis; hence, the presence of a carbon foundation is necessary in the growth media to compensate for the reduced carbon fixation [116]. Sucrose is most commonly used as carbon source in plant regeneration systems, being taken up from the growth medium by explants and hydrolyzed into glucose and fructose that further enter plant metabolism [117,118].

Apart from being utilized as energy source, sugars have important roles as signaling molecules. They are involved in the regulation of the activity of many genes and, through that, in many metabolic and developmental processes in plants, extending from embryogenesis up to senescence [119]. Three diverse sugar-sensing systems are in place: (1) hexokinase (HXK)-sensing system, (2) hexose transport-associated sensor (SUCROSE-NONFERMENTATION1-RELATED PROTEIN KINASE1 (SnRK1) and TARGET OF RAPAMYCIN (TOR) kinase) and (3) the Suc-specific pathway, which may implicate a signaling Suc transporter [120]. Germination, hypocotyl elongation, flowering, senescence, root and leaf growth, metabolism of carbon and nitrogen, pathogen attack and wounding are some of the processes that are shown to be influenced by glucose signaling via hexokinase-dependent pathways [121]. However, it was reported that AtRGS1, a regulator of G-protein signaling in *A*. *thaliana* might be implicated in the regulation of sugar responses during seedling development through an HXK-independent glucose signaling pathway [122].

Numerous reports have demonstrated the existence of crosstalk between phytohormone signaling and sugar sensing in higher plants, regulating developmental processes on transcriptional, posttranscriptional and posttranslational levels. Sugars can affect phytohormone response by altering the levels, localization and/or transport of different phytohormones [123,124,125].

Soluble sugars have been shown to be linked to auxin biosynthesis. When treated with glucose, *Arabidopsis* seedlings expressed multiple genes encoding auxin biosynthetic enzymes, including *YUC8* and *YUC9* [126]. The application of sucrose induced the expression of *YUC9* only in shoots and not in roots, even though the influence of sucrose on auxin levels was more prominent in roots compared to shoots, suggesting that auxin transport and conjugation pathways could be also affected by sucrose [127,128]. Conversely, sugar metabolism is also regulated by auxin signaling, as it was reported that the downregulation of the auxin response repressor *SlARF4* led to an increase in sugar and starch content in tomato [129]. The integration of sugar and auxin signals has also been shown on the morphogenic level through their joint regulation of *WOX7* in lateral root development [130].

Sugars and CKs play principal regulatory roles in plant growth and development acting agonistically, antagonistically or independently from each other on plant developmental responses. Transcriptome profiling of *Arabidopsis* seedlings treated with CKs and glucose revealed that the expression of an array of genes implicated in developmental pathways was altered, indicating a wide-ranging overlap between CK and glucose signal transduction [123,131]. The authors reported that, at the whole-genome level, 89% of genes were agonistically regulated by CK and glucose pathways, highlighting that the interaction between glucose and CKs plays a key and synergistic role in SAM activity. One of the agonistic interactions between sugar and CK was previously reported for the regulation of cyclin *CYCD3* expression, whereby sucrose presumably acted upstream of CK or synergistically with CK to regulate *CYCD3* expression [132,133,134]. The physiological outputs of such crosstalk need further investigation, as they may include changes in the cell cycle, photosynthetic activity, carbohydrate distribution and source/sink relations. Recently, it was proposed that de novo CK biosynthesis could be initiated via photosynthetically derived sugars when *Arabidopsis* seedlings were grown in high CO_2_ concentration [135]. The authors demonstrated the accumulation of CK precursors in shoots and roots prior to growth enhancement, and in roots, elevated expression of *IPT3* and *CYP735A2,* involved in de novo CK biosynthesis, was recorded [135]. These results confirmed previous reports of glucose and sucrose treatments upregulating CK biosynthesis [131,136].

A limited number of reports have discussed the relationship between phytohormone signaling, carbohydrate metabolism and shoot regeneration in cultured plant cells and tissues. The connection between shoot regeneration and starch metabolism was previously shown in sugarcane [137], tobacco [138], *Begonia rex* [139] and rice [140]. As for DNSO, particular attention has been paid to the role of sugars in callus induction, as genes involved in sugar metabolism are upregulated in this stage of organogenesis, presumably related to increased energy needs, but also to cell wall remodeling processes [48,141].

A role for sucrose uptake and metabolism in callus formation and shoot regeneration was demonstrated using immature rice seeds as starting material [118]. Genes involved in sucrose uptake and hydrolysis were upregulated on CIM, but also upon transfer to SIM. In contrast, starch content marked a sharp increase at day 7 on CIM and a sharp decline upon transfer to SIM, indicating that intense starch hydrolysis might be in place during shoot development. Endogenous levels of auxin, CK and abscisic acid (ABA) increased during incubation on CIM, as well as the expression of genes *OsPIN1*, *OsRR1* and *OsLEA1*, involved in auxin transport and CK and ABA signaling, respectively. The authors concluded that both phytohormone signaling and carbohydrate metabolism were tightly related to shoot organogenesis from rice calli.

Sugar-dependent callus development in *Arabidopsis* was shown to be regulated by the TOR-E2Fa module [47]. E2 PROMOTER-BINDING FACTOR a (E2Fa) is a cell-cycle-related transcription factor, previously reported as a key regulator of cellular pluripotency in plants. It activates S-phase cell cycle genes, enabling cell proliferation and the regulation of plant growth [142]. Using *HXK1*-deficient mutant *gin2-1* and estradiol-inducible *TOR*-RNAi *tor-es* plants, Lee and Seo [47] demonstrated that sugar-activated TOR kinase phosphorylates and increases the accumulation of E2Fa during callus formation and that TOR plays a role in the integration of sugar and auxin signaling, affecting E2Fa accumulation at post-translational level. Thus, regulation of this essential transcription factor by both sugar and auxin presumably facilitates the fine-tuned cellular competence for transdifferentiation and callus development in *Arabidopsis* [47].

Furthermore, the involvement of TOR kinase in the crosstalk between CK, light signaling and sucrose was shown in SAM formation [143]. Although crosstalk between these elements was shown in embryogenic SAM development, it is likely that the same, or similar interactions, take place during DNSO. The authors reported that the expression of *WUS* is light-dependent but photosynthesis-independent and involves CK signaling. Light-dependent *WUS* upregulation is mediated by Phytochrome B (PhyB), Cryptochrome 1 (Cry1), Cry2 and possibly PhyA, but is photosynthesis-independent, as it is unaffected by the presence of photosynthesis inhibitors. In addition, *WUS* expression could be induced in the dark, given the presence of sucrose in the media. Light intensity and sucrose showed additive effects on *WUS* induction, suggesting that these two factors induce *WUS* independently of each other. Both light-dependent and sucrose-dependent signaling pathways are likely to employ CK signalization. Additionally, CK signaling is actively inhibited in the dark through the activity of cytokinin oxidases/dehydrogenases, CKX5 and CKX6. The results suggested that sucrose merely represents a nutrient and not a signal in *WUS* activation, as palatinose, a non-nutritive sucrose analog otherwise capable of triggering sucrose-dependent signaling, was shown to be ineffective. Both the light-dependent and sucrose-dependent *WUS* activation employed TOR kinase, mediating crosstalk between CK, light signaling and the metabolic status of the plant [143].

Further indication of crosstalk between sugar and phytohormones in DNSO recently came from our research group [144]. Significant influence of both CK and sucrose treatment, as well as their interaction, was observed during various stages of DNSO from kohlrabi seedlings. Results demonstrated a remarkable increase in endogenous CK levels when 2 mg L^−1^ CK *trans*-zeatin (*t*Z) and high sucrose concentration (9%) were applied together, suggesting that sucrose may interact with CK uptake and/or homeostasis. In addition, higher concentration of sucrose significantly affected organogenesis-related genes involved in auxin transport, CK response, SAM formation and cell division, while correlation analysis suggested that sucrose could affect endogenous CK levels and their impact on the transcriptional activity of analyzed genes during callus and shoot formation in kohlrabi shoot organogenesis [144].

Taken together, these reports offer only partial insight into a highly complex network of phytohormone and sugar signaling, which underlies the balanced regulation of callus formation and shoot regeneration. The case of sucrose, which is routinely added to the regeneration media as a carbon source and was never intended to interfere with shoot regeneration, suggests that any component of nutrient media, just like any factor in general present in the environment of a cultured plant, can interact with the signaling pathways that regulate shoot regeneration or any other process being studied in cultured plants.

## 5. Hormone Uptake: The Missing Link for the Integrative Interpretation of DNSO

Ever since the first protocols for shoot regeneration were developed, their efficiency in inducing callus formation and shoot regeneration were interpreted in relation to the composition of plant growth regulators. However, in an in vitro shoot organogenesis system, the hormonal composition of CIM and SIM represents only the first input parameter in the complex regeneration process. The output, in terms of shoot regeneration efficiency, is the resultant of hormone uptake by the explants, transport to target tissues, alterations in hormonal homeostasis, and downstream signaling processes leading to shoot regeneration. Thus, interpreting shoot regeneration efficiency as a simple output of the media composition represents a rough, albeit often practical, simplification.

Of the several steps linking the hormonal composition of the media to the final shoot regeneration output, the first one—hormone uptake—is the most neglected and the least studied. DNSO occurs from a callus mass and/or a starting tissue explant of various origin (root, hypocotyl, cotyledon, etc.) that is in contact with the regeneration medium. The mechanisms of hormone uptake from the regeneration medium have not been studied sufficiently, and nothing is known about the differences in the mechanisms of uptake between tissues of various origin. However, it is conceivable that these mechanisms are “borrowed” from other developmental programs; thus, the uptake of growth regulators from the nutrient media is presumably carried out similarly to the hormone uptake from the intercellular spaces within an intact plant. For auxin, uptake likely relies on hormone transporters, membrane-associated proteins responsible for phytohormone transport and uptake within the plant body. For CK, the term “uptake” has a broader meaning. If CK signaling occurred entirely from the plasma membrane, CK molecules would not need to be taken up by the cells; however, they would still need to join the pool of apoplastic CK to be available to their target cells for signalization. Hence, CK “uptake” by the explant in the broader sense can be considered as the moment when an exogenous growth regulator becomes indistinguishable from the endogenous phytohormone. Additionally, recent research has suggested the importance of ER-located CK signalization, implying that the intracellular uptake of CK is also an essential step in CK perception [145,146].

In this section, we provide a brief overview of the known auxin and CK transporters that operate in plant growth and development, including the regulation of DNSO. Some of these transporters might be responsible for hormone uptake from the regeneration media during DNSO, whereas all of them take part in subsequent transport of auxin and CK through the cultured plant tissues.

### 5.1. The Role of Auxin Transport

Auxin is transported through the plant body by two distinct mechanisms: fast, non-directional auxin transport through the phloem and slow, directional polar auxin transport (PAT) between neighboring cells [147]. Both mechanisms consist of auxin transporters, localized on the plasma membrane (PM), endoplasmic reticulum (ER) or vacuolar membrane. In general, auxin transporters are divided into influx and efflux carriers, depending on whether they mediate the uptake of auxin into the cell or its export from the cell [147,148].

#### 5.1.1. Members of the PIN Family Are Auxin Efflux Carriers Responsible of Polar Auxin Transport

Many developmental processes in plants require local auxin concentration gradients [147]. Such local auxin gradients are also crucial at various stages of DNSO, such as founder cell specification, the induction of the pluripotent primordium and the establishment of *CUC*- and *WUS*-expressing domains for the formation of the SAM (reviewed in [4] and earlier in this review). These local auxin gradients are formed through the local, CK-induced biosynthesis of auxin [35], along with PAT, operating between neighboring cells and relying on auxin efflux carriers of the PIN-FORMED (PIN) family [147,149].

There are eight PIN transporters in *Arabidopsis* (PIN1-PIN8), which share a similar structure with conserved hydrophobic N- and C-terminal domains, separated by a central, hydrophilic loop of variable length. “Canonical” or “long” PINs (PIN1-PIN4 and PIN7) are localized on the PM and involved in direct, polar intercellular auxin transport, whereas “short” PINs (PIN5, PIN6 and PIN8) are localized primarily on the ER and play a role in intracellular auxin distribution [147,149]. However, the “short” PINs can also be localized to the PM [150,151]. According to the chemiosmotic model for efflux carriers, PINs display a polar subcellular localization, related to the direction of auxin flow [152]. PIN proteins undergo constitutive endocytic recycling, being quickly rearranged between different sides of the cell, enabling the prompt redirection of auxin flux in response to adequate signals [153].

*PIN* genes display tissue- and developmentally specific expression patterns, enabling individual members of the gene family to regulate various aspects of plant development through auxin redistribution [154,155]. Interestingly, although all “long” PINs mediate the efflux of IAA, their capability to transport other auxins varies. Thus, PIN2 and PIN7 mediate the efflux of 2,4-D [156], whereas PIN4 and PIN7 can mediate the efflux of NAA [157,158]. “Short” PINs mediate the trafficking of both IAA and NAA between the cytoplasm and the ER [155]. These differences in substrate specificity between different PIN family members might be partly relevant to the variations in plant responses to the application of different auxins in tissue culture.

Since PIN transporters mediate the polar distribution of auxin through facilitating auxin efflux, the asymmetric, polar distribution of “long” PIN proteins on the PM determines the direction of auxin flow between cells. Hence, the proper spatial distribution of PINs on the cell membrane is necessary for the establishment of the correct auxin polarity. The spatial distribution of PINs is regulated mainly through the balance between PIN phosphorylation and dephosphorylation, mediated by specific kinases and phosphatases [159,160]. PIN phosphorylation is mediated by at least three subclades of AGC kinases, namely: (1) PINOID (PID) kinase and its close homologs, WAVY ROOT GROWTHs (WAGs); (2) D6 PROTEIN KINASE (D6PK) and D6 PROTEIN KINASE-LIKEs (D6PKLs) and (3) PROTEIN KINASE ASSOCIATED WITH BRX (PAX) and PAX-LIKE (PAXL) [160]. The dephosphorylation of PINs is mediated by PROTEIN PHOSPHATASE 2A, (PP2A, [161]), PP4 [162] and PP6 [163], which are in antagonistic balance with PID and other PIN kinases, necessary for the establishment of correct PIN polarity. Additional regulation of the polar distribution of PINs is provided by MACCHI-BOU4/ENHANCER OF PINOID/NAKED PINS IN YUCCA-LIKE1 (MAB4/ENP/NPY1), possibly through the enhancement of PID activity [164,165], and by RCC1-LIKE DOMAIN (RLD) proteins [166].

At the transcriptional level, *PIN* genes are regulated by an array of transcription factors in response to various stimuli, including XAANTAL2 [167], INDETERMINATE DOMAIN 14/15/16 (IDD14/15/16) [168] and even an auxin response factor, ARF5/MP thus providing direct regulation of auxin transport by auxin itself [37,169]. The activity of PINs is also regulated by virtually all other phytohormone cascades, including CK, ABA, gibberellin, jasmonic acid, salicylic acid, strigolactones and brassinosteroids [160].

#### 5.1.2. ABCB Transporters Mediate Non-Directional Auxin Transport and Interact with PIN Carriers

Beside PINs, auxin efflux is mediated through the ATP-BINDING CASSETTE-B/P-GLYCOPROTEIN (ABCB/PGP) transporters [155,170]. Not all ABCB transporters are efflux carriers; for instance, ABCB4 has been shown to function both as an influx carrier [171,172] and efflux carrier [173], whereas ABCB21 is a facultative influx or efflux carrier depending on intracellular auxin concentration [174]. Mutations in *ABCB* genes lead to defective phenotypes, indicating essential roles in plant development [170]. Transport of IAA is mediated by ABCB1, ABCB4, ABCB19 and ABCB21, whereas ABCB1 and ABCB19 are capable of additionally transporting 2,4-D, while ABCB4 mediates the transport of IAA, 2,4-D and NAA [156,171,173,175,176].

In contrast to the PINs, ABCB-mediated auxin efflux is not polar, and instead it functions to fine-tune the amount of auxin available for directional transport. However, ABCB transporters are capable of modulating the activity of PINs through direct interaction [170]. Interestingly, ABCBs and PINs undergo joint regulation by PID kinase [177], as well as by plant secondary metabolites, flavonols [178].

#### 5.1.3. AUX1/LAX Transporters Are Auxin Influx Carriers

Auxin influx is mediated by the members of the AUXIN-RESISTANT1/LIKE-AUX1 (AUX1/LAX) family, H+ symporters located at the PM: AUX1, LAX1, LAX2 and LAX3. Mutations in corresponding genes confer specific defective phenotypes, as each of these auxin influx carriers plays specific roles in developmental processes, including photo- and gravitropism, apical hook development and the development of lateral roots and root hair [148,179]. AUX1 plays a role in the establishment of local auxin maxima in the xylem pole of the pericycle, necessary for the specification of the founder cells of the LRP or pluripotent primordia in organogenesis [4,34]. The expression of LAX3 genes in cortical cells is induced by the auxin from the LR primordia. This accumulation of auxin in cortical cells leads to the induction of cell wall remodeling enzymes necessary for the penetration of the growing primordium through the outer cortex of the primary root [180]. Due to the developmental equivalence between LRP and the pluripotent primordium in organogenesis, similar processes occur during the corresponding phases of DNSO [48,49].

As for substrate specificity, AUX1 was shown to mediate the uptake of both IAA and 2,4-D but not NAA and indole-3-butyric acid (IBA) [181,182]. Similarly, LAX1 and LAX3 mediate the influx of IAA and 2,4-D but not NAA [180,183].

#### 5.1.4. Other Auxin Transporters Include NRT1.1, PILS and WAT1

Beside PINs, ABCBs and AUX1/LAX, other known auxin transporters include: NITRATE TRANSPORTER1.1 (NRT1.1), PIN-Like Transporters (PILS), and WALLS ARE THIN1 (WAT1).

NRT1.1 is a nitrate transporter that also mediates auxin uptake, thus integrating the hormonal response with nutrient availability [184,185,186,187,188]. NRT1.1 is also responsible for the basipetal transport of auxin, driving it away from the tips of LRPs. Notably, NRT1.1-mediated auxin uptake is inhibited by high nitrate concentrations [184]. Furthermore, local auxin biosynthesis in the root stele is downregulated by NRT1.1 but enhanced upon nitrate flow through NRT1.1 [189]. The relevance of such crosstalk between auxin and nitrogen in in vitro culture, where nitrate is importantly supplied through the nutritive media, is another point where media components possibly interact with DNSO, and thus might be worth exploring in the future.

Two of the PILS transporters, PILS2 and PILS5, have been shown to play a role in the regulation of intracellular auxin accumulation, presumably through its sequestration in the ER. *PILS2* and *PILS5* negatively regulate auxin signaling, as shown with the development of lateral roots—single *pils2* and *pils5* and double *pils2pils5* mutants developed an increased number of lateral roots, whereas a *pils5* gain-of-function mutant was impaired in lateral root formation [190]. By facilitating the compartmentalization of the intracellular auxin to the ER, the PILS transporters decrease the amount of intracellular auxin available for the migration to the nucleus and the subsequent signaling. More recent research has shown that, at least in some developmental processes, PILS might be controlled by PhyB, suggesting crosstalk between light signaling and auxin transport [191].

The latest auxin transporter to be discovered has been a vacuolar transporter WAT1, with a likely role in vacuolar storage of auxin [192].

#### 5.1.5. How Do Auxin Transporters Affect the Auxin Uptake in DNSO?

Auxin transporters have a dual role in DNSO. Firstly, they provide highly coordinated regulation to multiple cellular and developmental processes underlying various phases of DNSO through the formation of local auxin maxima and minima, which are necessary for the establishment of expression domains for crucial morphogenic genes regulating organogenesis. Secondly, they represent the first molecular mechanism through which a cultured explant establishes a contact with the growth regulators present in the regeneration media. Throughout DNSO, as well as during other morphogenic processes taking place in in vitro culture, this first step defines and affects the further “molecular journey” of an exogenous growth regulator being taken up in the plant tissue—i.e., it can, upon uptake, either stay in the first cell where it was taken up and start a signaling cascade, undergo metabolic changes and/or degradation, or be further transported to neighboring or more distant target cells where it will perform its physiological output. This path largely depends on the nature of the transporters present on the outer surface of the explant, including their substrate specificity, stability, intracellular trafficking patterns, compartmentalization, etc.

Thus, when trying to evaluate the relevance of particular auxin transporters for the process of DNSO, influx carriers represent likely candidates for the uptake of auxin from the media, whereas the further fate of the uptaken auxin depends on the compartmentalization of these carriers and their connection with the next steps in the molecular path of the uptaken hormone. It is likely that AUX1/LAX influx carriers might be responsible for the initial uptake of auxin from the media, as it was reported that the root explants of *aux1* mutants could not form calli under typical culture conditions; however, elevated concentration of auxin in CIM led to initiation and formation of calli, as well as subsequent shoot regeneration [31]. The capability of *aux1* mutants to form calli on media with high auxin concentration suggests that auxin influx through passive diffusion and/or additional importers, although occurring at a lesser rate, can contribute to callus formation. Thus, the initial uptake of exogenous auxin from the media by AUX1 is a promising hypothesis, but it needs to be further tested. Moreover, the subsequent subcellular fate of the uptaken exogenous molecule needs to be clarified, especially considering the emerging importance that subcellular compartmentalization plays in phytohormonal homeostasis and regulation [193].

Recently, a novel genetically encoded biosensor for the in vivo imaging of auxin gradients with a high spatial and temporal resolution was developed based on the bacterial tryptophan repressor (TrpR) [194]. Ten minutes after treating transgenic *Arabidopsis* seedlings with IAA, the signal in root nuclei reached a maximum, after which it remained constant for another 50 min. A blank control, consisting of the solvent dimethyl sulfoxide, did not induce a response. Additional measurements within shorter intervals suggested that the uptake of the auxin from the extracellular space was a fast, highly efficient and constitutive process; however, efflux from the cell was slower and restrictive [194]. This study represents the first step towards tracking and understanding the “real”, in situ pathway of exogenous auxin molecules on their journey through the plant body.

Lastly, the role for auxin conjugation in plant tissue culture needs to be clarified. The conjugation of auxin to a wide range of molecules, such as amino acids, sugars or even proteins and polysaccharides, is known to occur as a mechanism of hormonal homeostasis [195]. It has been demonstrated that the synthetic auxin 2,4-D is conjugated to aspartate in cultured *Arabidopsis* seedlings and that the 2,4-D-Asp conjugate is hydrolyzed to a free auxin at a substantially higher rate compared to IAA-Asp [196]. The possibility for plants to metabolize a synthetic auxin that is used for callus induction is potentially relevant to the understanding of DNSO and should be taken into account when interpreting the molecular pathway of auxin upon its uptake from the media.

### 5.2. The Role of Cytokinin Transport

During DNSO, CK from the regeneration media needs to be taken up in the plant tissues to exert its physiological effects. CK uptake is considered to occur rapidly; however, various traits of the plant tissue, collectively addressed as “competence for regeneration”, determine the ability of the tissue to respond to the applied hormone [197,198]. Genotype, explant type and age are some of the factors affecting the regeneration competence, presumably mediated by variations in the efficiency of CK uptake, tissue sensitivity to CK or even the abundance of totipotent cells. For instance, young and immature explant tissues often express greater regeneration potential, which is ascribed to their richness in stem cells [6].

Indirect evidence of CK uptake by explants in in vitro culture is abundant. When exogenous CK from the nutrient media is being taken up by in vitro grown plants or plant tissues, it directly affects endogenous CK levels. Additionally, an uptake-driven increase in endogenous CK is usually accompanied by CK *O*- and/or *N*-glucosylation, as deduced from a concomitant increase in these conjugates [108,144,198,199,200]. Conjugation is a common mechanism by which plants achieve hormonal homeostasis through the quick removal of excess bioactive CK [195]. The conjugation of CK molecules, particularly by *N*-glucosylation, has been long considered an irreversible mechanism of CK inactivation. It was, however, recently shown that CK *N*-glucosides do not always lack biological activity [201,202,203,204], although predominantly due to their metabolic conversion resulting in the release of active CK nucleobases [205,206]. Besides altering endogenous CK, the uptaken CK may also affect other endogenous phytohormones, with changes in endogenous auxin being the most relevant to the process of organogenesis [31,32,108].

Particular mechanisms of CK uptake by explants during DNSO have not been described, but CK uptake and transport through explant tissues can be expected to rely on CK transporters. The currently known CK transporters fall into four categories: PURINE PERMEASES (PUPs), EQUILIBRATIVE NUCLEOSIDE TRANSPORTERS (ENTs), ATP-BINDING CASSETTE-G (ABCG) and AZA-GUANINE RESISTANT (AZG) transporters [207,208].

#### 5.2.1. PUP and AZG Transporters Function as Importers of CK Nucleobases

PUPs are transmembrane proteins with affinity for a wide array of nucleobases and adenine-based molecules, such as adenine, CK nucleobases and even adenosine and caffeine [209]. In the *Arabidopsis* genome, 23 *PUP* genes were found, differing in tissue-specific expression and possible physiological roles [208]. In a yeast expression system, AtPUP1 could mediate the uptake of adenine, *t*Z and iP; AtPUP2 was able to uptake adenine, iP, kinetin and benzyl adenine (BA), whereas uptake of both *trans*- and *cis*-zeatin was less efficient; in the same expression system, AtPUP3 was ineffective at adenine uptake [209].

Recently, a unique role was reported for AtPUP14 in the embryogenic development of *Arabidopsis* [210]. The widespread expression of AtPUP14 on the PM, which topped the expression of all other AtPUP family members, caused intense cellular uptake of extracellular CK, thereby depleting the apoplastic CK pool and downregulating CK signaling at the PM of its target tissues. The constitutive expression of *AtPUP14* from the 35S promoter was lethal. Thus, the intensity of the CK signal was inversely correlated with *PUP14* expression, thereby revealing a peculiar mechanism of regulation of CK signaling. Radiolabeling and competition assays showed that *t*Z was a substrate for AtPUP14; the uptake of *t*Z could be competitively inhibited by iP and BA but not by *t*Z riboside (*t*ZR) [210].

The rice PUP family has 13 members (OsPUP1-OsPUP13), of which OsPUP1, OsPUP4 and OsPUP7 function as CK transporters. Interestingly, only OsPUP4 localizes to the PM, whereas OsPUP1 and OsPUP7 localize to the ER [211,212].

Recently, it was reported that CK membrane transport could be also facilitated by two members of the AZG family, AtAZG1 and 2, which act as adenine and guanine importers [213,214]. Thus, being nucleobase importers, AZG transporters are functionally similar to PUPs. AtAZG1 interacts with PIN1 and stabilizes it on the plasma membrane in *Arabidopsis* root cells, together with AtAZG2. Hence, it was suggested that AtAZG1 controls root cell architecture through modulating the intracellular auxin/CK ratio [215]. Furthermore, AtAZG2, correspondingly to AtPUP14, was also suggested to alter CK distribution, possibly for the regulation of root architecture. AZG2 enables membrane transport of CK nucleobases in both directions in yeast cells. Although AZG1 is localized only at the PM, AZG2 is localized at both the PM and the ER [214].

#### 5.2.2. ENTs Are Importers of CK Nucleosides

Whereas PUPs primarily function as importers of CK nucleobases, ENTs are importers of nucleosides, which are widely regarded as primary CK transport forms. In *Arabidopsis*, eight *ENT* genes were identified, whereas in rice, there are only four. Members of the family are variable with regards to tissue specificity and the mechanism of transport. Furthermore, *t*ZR and iPR were suggested as the most important substrates for *ENT*-mediated transport of CK nucleosides, whereby root-derived *t*ZR was supposed to be taken up from the xylem in the above-ground parts and shoot-derived iPR was transported basipetally and taken up from the phloem by root cells [216,217,218]. The basipetal transport of iP-type CKs from shoot to root has been shown to occur through symplastic connections in the phloem [219], but no evidence has been presented of its mechanism, which, as of today, remains unknown [207].

#### 5.2.3. Long-Distance Transport of Trans-Zeatin through Xylem Is Mediated by the ABCG14 Transporter

A crucial role in the long-distance transport of *t*Z-type CKs through the xylem has been shown for a CK export transporter, ABCG14 [220,221]. The loss of function of the *ABCG14* gene, which is predominantly expressed in the root stele and pericycle, caused the depletion of *t*Z-type CKs in *Arabidopsis* shoots, suggesting their delivery to the shoot through the xylem. The predominance of *t*ZR as the main molecular form of CK transported through the xylem has been reported several times, highlighting the importance of this molecule for long-distance signaling [218,222,223]. Interestingly, intracellular CK uptake by the root cells has been suggested as a mechanism modulating the amount of CK available for long-distance transport to the shoot; both PUPs and ENTs are hypothesized to underlie this secondary uptake [224].

#### 5.2.4. CK Movement during DNSO: Where from, Where to, and What for?

In tissue culture, CKs delivered acropetally to the regenerating shoots originate mainly from the regeneration media. Root-borne CKs may be additionally present if the regenerating explants contain roots or root segments, but even then, the CK from the media contributes to the bulk of CK delivered to the shoot.

Accumulating evidence points at novel models that challenge traditional concepts of CK transport and perception. New insights into CK perception suggest a relevance of both ER-based and PM-based CK signaling in plants [145,146]. Furthermore, multiple reports on the presence of purine transporters on the ER [211,212,214] have opened new possibilities for the interpretation of CK flow through plant tissues. According to the proposed interpretation, the lumen of the ER represents a novel domain for CK action, equipped with elements of CK perception, metabolism, transport and signaling that are equivalent but not identical to the ones present in the apoplast. Hence, CK should be able to circulate through the symplast [225]. As compartmentalization is becoming an increasingly important aspect of phytohormone action [193], questions are raised regarding the differences between CK signals delivered in different compartments. An interesting interpretation of dual CK action was recently proposed, suggesting the co-existence of two complementary CK signals governed by different dynamics—the rapid and intense “Hulk” system, mediated by signaling components and metabolic enzymes that are quickly upregulated to a great amplitude but with temporary effects, and the low-amplitude but constitutively active “Deadpool” system. Such a two-channel system of CK action would enable the efficient fine-tuning and synchronization of the multitude of heterogenous actions that need to be simultaneously processed through the CK signaling cascade [226].

Whether the two domains of CK transport and perception represent a “Hulk” and a “Deadpool” or have some other meaning in the regulation of CK action, we need to consider that this aspect of CK compartmentalization also affects the CK molecules originating from the regeneration media. Thus, as soon as an exogenous CK molecule arrives in the proximity of the cultured explant via diffusion through the regeneration medium, it may join one of the compartmentalized CK domains within the explant tissue, which determines its further molecular path and ultimately, its output on organogenesis. The identity of other components of this pathway, e.g., the particular receptor binding the CK molecule, might also affect the nature of this output. Hopefully, in the following years, knowledge on the functional significance of different CK signaling domains will increase, providing also the possibility for more comprehensive interpretation of the fundamental role of CK in organogenesis.

## 6. Conclusions and Future Perspectives

In the last 30 years, the basis for our understanding of DNSO has evolved from an empirical to a sophisticated theoretical framework, explained in detail on a molecular level. However, it appears that the research on molecular regulation of DNSO has been focused too tightly on its developmental aspects, commonly disregarding elements of the context in which DNSO is taking place, such as the interactions between the cultured explant and its immediate environment—the regeneration medium.

In an integrative approach to the interpretation of DNSO, the pathway of plant growth regulators as initiators of DNSO needs to be considered in its entirety, from their uptake from the regeneration medium and transport to their target cell through their effect on plant hormonal homeostasis and eventually, to the resulting signaling outputs. Until recently, all steps of this pathway have not received appropriate attention from researchers. Research on hormone uptake from nutrient media by plant tissues is particularly scarce. For a comprehensive interpretation of DNSO, hormone uptake needs to be thoroughly investigated, and the relationship between (exogenous) growth regulator application and (endogenous) plant hormone homeostasis theoretically explained. After decades of routine application of DNSO, we cannot continue explaining the effects of exogenous growth regulators on shoot regeneration simply by their presence in the regeneration media.

The same integrative approach needs to consider all the environmental factors surrounding the regenerating explant and provide a comprehensive explanation for their interference with the hormonal control of organogenesis. For instance, there is cumulative evidence that sucrose, a common component of regeneration media, affects shoot regeneration through crosstalk with auxin and CK signaling and possibly, when present at higher concentration, even interfering with hormone uptake from the media.

The central role of plant hormones as integrators of environmental cues and mediators of the modifications of plant growth and development in response to those cues implies great sensitivity to all elements that are present in the environment of the cultured plant. Assuming that a complex developmental program, such as DNSO, is regulated by auxin and CK, we can expect all the environmental factors present in the tissue culture to affect that regulation. Thus, a comprehensive explanation of the hormonal regulation of DNSO is impossible without taking all those environmental factors into account, and the future of the shoot regeneration technology is unimaginable without such integration.

## Figures and Tables

**Table 1:** A summary of genes involved in auxin and cytokinin transport and in de novo shoot organogenesis (DNSO). A brief explanation for the function of particular genes and their involvement in the stages of DNSO is provided.

Gene Abbreviation	Full Name	Function	Relevant Stage(s) of DNSO
*ABCB*	*ATP-BINDING CASSETTE B*	auxin efflux carrier (non-polar transport)	probably all stages of DNSO
*ABCG*	*ATP-BINDING CASSETTE G*	cytokinin exporter	unclear
*AHK*	*ARABIDOPSIS HISTIDINE KINASE*	cytokinin receptor	multiple stages of DNSO
*AHP*	*ARABIDOPSIS HISTIDINE PHOSPHOTRANSFER*	cytokinin signaling	multiple stages of DNSO
*AP2/ERF*	*APETALA2/ETHYLENE RESPONSE FACTOR*	class of transcription factors (includes: *PLT*, *WIND*, *ESR*)	multiple or all stages of DNSO
*ARF*	*AUXIN RESPONSE FACTOR*	auxin signaling (transcription factor)	all stages of DNSO
*ARR-A*	*ARABIDOPSIS RESPONSE REGULATOR* (type-A)	cytokinin signaling (negative regulator)	multiple stages of DNSO
*ARR-B*	*ARABIDOPSIS RESPONSE REGULATOR* (type-B)	cytokinin signaling (transcription factor)	multiple stages of DNSO
*AS*	*ASYMMETRIC LEAVES*	transcription factor	multiple stages of DNSO
*Aux/IAA*	*AUXIN/INDOLE-3-ACETIC ACID*	auxin signaling (repressor)	all stages of DNSO
*AUX1*	*AUXIN-RESISTANT1*	auxin influx carrier	founder cell specification and primordium formation; possibly other stages
*AZG*	*AZA-GUANINE RESISTANT*	cytokinin nucleobase importer	unclear
*bZIP59*	*basic region LEUCINE ZIPPER59*	transcription factor	CIM-induced primordium formation
*CKX*	*CYTOKININ OXIDASE/DEHYDROGENASE*	cytokinin catabolism	multiple stages of DNSO
*CLV*	*CLAVATA*	transcriptional regulator	acquisition of shoot identity
*Cry*	*CRYPTOCHROME*	light perception	acquisition of shoot identity
*CUC*	*CUP-SHAPED COTYLEDON*	transcription factor	multiple stages of DNSO
*CYCD3*	*CYCLIN D3*	cell cycle regulation	multiple or all stages of DNSO
*E2Fa*	*E2 PROMOTER BINDING FACTOR a*	cell cycle-related gene	primordium initiation; possibly other stages
*ENT*	*EQUILIBRATIVE NUCLEOSIDE TRANSPORTER*	cytokinin nucleoside importer	unclear
*ESR* (*DRN*, *DRNL*)	*ENHANCER OF SHOOT REGENERATION* (*DORNRÖSCHEN, DORNRÖSCHEN-LIKE*)	transcription factor	multiple stages of DNSO
*FAD-BD*	*FAD-BINDING BERBERINE*	cell wall metabolism	CIM-induced primordium formation
*GATA23*	*GATA-MOTIF BINDING*	transcription factor	founder cell specification (lateral root)
*HAG1*	*HISTONE ACETYLTRANSFERASE-GNAT SUPERFAMILY1*	histone acetyltransferase (epigenetic regulation)	CIM-induced primordium initiation
*HSFB1*	*HEAT SHOCK FACTOR B1*	transcription factor	wound-induced callus formation
*HXK*	*HEXOKINASE*	sugar metabolism and signaling	unclear
*IPT*	*ISOPENTENYL-TRANSFERASE*	cytokinin biosynthesis	multiple stages of DNSO
*KRP*	*KIP-RELATED PROTEIN*	inhibitor of cyclin-dependent kinase	multiple or all stages of DNSO
*LAX*	*LIKE-AUX1*	auxin influx carrier	founder cell specification and primordium formation; possibly other stages
*LBD/ASL*	*LATERAL ORGAN BOUNDARIES DOMAIN/ASYMMETRIC LEAVES2-LIKE*	transcription factor	CIM-induced primordium formation
*LOG*	*LONELY GUY*	cytokinin biosynthesis	multiple stages of DNSO starting from primordium formation
*NRT1.1*	*NITRATE TRANSPORTER1.1*	nitrate uptake, auxin uptake	probably the initial stages of DNSO
*PHB*	*PHABULOSA*	transcription factor	acquisition of shoot identity
*PHV*	*PHAVOLUTA*	transcription factor	acquisition of shoot identity
*Phy*	*PHYTOCHROME*	light perception	acquisition of shoot identity
*PID*	*PINOID*	PIN kinase	all stages of DNSO
*PILS*	*PIN-LIKE TRANSPORTERS*	auxin transport through the ER membranes	CIM-induced primordium formation; possibly other stages
*PIN*	*PIN-FORMED*	auxin efflux carrier (polar transport)	all stages of DNSO
*PLT*	*PLETHORA*	transcription factor	multiple or all stages of DNSO
*PP*	*PROTEIN PHOSPHATASE*	PIN dephosphorylation	all stages of DNSO
*PUP*	*PURINE PERMEASE*	cytokinin nucleobase importer	unclear
*REV*	*REVOLUTA*	transcription factor	acquisition of shoot identity
*SCR*	*SCARECROW*	transcription factor	primordium initiation; possibly other stages
*SnRK1*	*SUCROSE-NONFERMENTATION1-RELATED PROTEIN KINASE1*	sucrose signaling	unclear
*STM*	*SHOOTMERISTEMLESS*	transcription factor	acquisition of shoot identity
*TIR1/AFB*	*TRANSPORT INHIBITOR RESPONSE1/AUXIN SIGNALING F-BOX*	auxin receptor	all stages of DNSO
*TOR*	*TARGET OF RAPAMYCIN*	sucrose signaling	at least in CIM-dependent primordium formation
*WAT1*	*WALLS ARE THIN1*	auxin transporter (vacuolar)	unclear
*WIND*	*WOUND-INDUCED DEDIFFERENTIATION*	transcription factor	wound-induced callus formation
*WOX*	*WUSCHEL-RELATED HOMEOBOX*	transcription factor	all stages of DNSO
*WUS*	*WUSCHEL*	transcription factor	acquisition of shoot identity
*YUC*	*YUCCA*	auxin biosynthesis	multiple or all stages of DNSO

## Data Availability

Not applicable.
